# Lysine-specific demethylase 3A (KDM3A) protects against ovarian dysfunction in premature ovarian insufficiency by transcriptional activation of ATP-binding cassette sub-family A member 7 (ABCA7)-mediated mitochondrial homeostasis

**DOI:** 10.1186/s43556-026-00567-5

**Published:** 2026-09-08

**Authors:** Xiangrong Cui, Chongyang Han, Puhua Zhang, Ruixiang Zhu, Li Peng, Jiali Luo, Chenyu Jia, Ruotong Ju, Xinyu Zhu, Shu Wang, Huihui Li, Xiaochun Liu, Xuan Jing

**Affiliations:** 1 Shanxi Bethune Hospital, Shanxi Academy of Medical Sciences, Tongji Shanxi Hospital, Third Hospital of Shanxi Medical University, Taiyuan, 030032 China; 2 Department of Reproductive Medicine Center, Children’s Hospital of Shanxi, The Affiliated Children’s Hospital of Shanxi Medical University, Shanxi Maternal and Child Health Hospital, Taiyuan, 030001 China; 3 Department of Clinical Laboratory of Shanxi Provincial People’s Hospital, Shanxi Medical University, Taiyuan, 030001 China; 4 Shanxi University of Traditional Chinese Medicine, Taiyuan, Shanxi 030001 China; 5 Department of Nephrology of Shanxi Provincial People’s Hospital, Shanxi Medical University, Taiyuan, 030001 China

**Keywords:** KDM3A, Premature ovarian insufficiency, ABCA7, Mitochondrial homeostasis, Granulosa cells

## Abstract

Premature ovarian insufficiency (POI) is a heterogeneous disorder characterised by accelerated follicular depletion and ovarian dysfunction before age 40, yet effective therapeutic strategies remain limited. Here we identify the histone demethylase KDM3A (lysine‑specific demethylase 3A) as a critical protective factor in POI. We found that KDM3A expression is significantly downregulated in both granulosa cells from idiopathic POI patients and a cisplatin‑induced POI mouse model, and its levels correlate positively with serum AMH and oestradiol and negatively with FSH and LH. KDM3A‑deficient mice exhibited exacerbated cisplatin‑induced ovarian failure, with increased follicular atresia, elevated apoptosis, and hormonal imbalances, whereas ovarian‑specific KDM3A overexpression restored follicular development, oocyte quality, hormonal balance, and fertility. Mechanistically, chromatin immunoprecipitation sequencing identified ATP‑binding cassette sub‑family A member 7 (ABCA7) as a direct transcriptional target of KDM3A. KDM3A binds to the ABCA7 promoter, removes the repressive H3K9me2 mark, and activates ABCA7 transcription. KDM3A‑mediated ABCA7 upregulation preserves mitochondrial integrity, reduces oxidative stress, maintains mitochondrial membrane potential and ATP production, and suppresses granulosa cell apoptosis. Notably, ABCA7 overexpression alone phenocopied the protective effects of KDM3A, indicating that ABCA7 acts as a key downstream effector. Collectively, our findings uncover a previously unrecognised KDM3A‑ABCA7 epigenetic axis that safeguards mitochondrial homeostasis in granulosa cells. This pathway not only advances our understanding of POI pathogenesis but also provides potential molecular targets for therapeutic intervention in chemotherapy‑induced ovarian injury.

**Supplementary Information:**

The online version contains supplementary material available at https://doi.org/10.1186/s43556-026-00567-5.

## Introduction

The ovary is a pivotal reproductive and endocrine organ, playing a crucial role in mammalian fertility and sexual function [1–3]. Premature Ovarian Insufficiency (POI) is a debilitating clinical disorder characterized by the loss of ovarian follicular reserve before the age of 40, leading to amenorrhea, hypergonadotropism, and estrogen deficiency [4–6]. This condition not only results in infertility but also increases long-term health risks, including osteoporosis and cardiovascular diseases [7–9]. The pathogenesis of POI is heterogeneous, involving genetic, autoimmune, and iatrogenic factors; however, the underlying molecular mechanisms remain incompletely understood, and effective interventions to halt or reverse ovarian function decline are limited [10–12].

Mounting evidence underscores the central role of mitochondrial dysfunction in the pathogenesis of POI [13–15]. As the cellular powerhouses, mitochondria are indispensable for providing the vast energy required for oocyte maturation, follicular development, and steroid hormone synthesis [16–18]. Beyond energy production, mitochondria are key regulators of calcium metabolism, redox signaling, and, critically, the intrinsic pathway of apoptosis [19–21]. Granulosa cell apoptosis is a hallmark of POI, and mitochondrial-controlled cell death pathways have been strongly implicated in the accelerated follicle atresia observed in this condition [5, 17, 22]. Thus, identifying regulators that preserve mitochondrial homeostasis in ovarian cells represents a promising therapeutic strategy for POI.

Epigenetic modifications, particularly histone methylation, have emerged as critical regulators of gene expression in various physiological and pathological processes [23–25]. However, while the role of histone methylation in POI is gaining attention, the function of histone demethylation is comparatively less explored [26]. Lysine-specific demethylase 3 A (KDM3A, also known as JMJD1A), specifically removes the repressive histone mark H3K9me2, thereby activating the transcription of target genes [27–30]. Previous studies have demonstrated that KDM3A plays a vital role in metabolic and vascular diseases [31–33]. For instance, KDM3A-mediated H3K9me2 demethylation is crucial in diabetic vascular remodeling, and its benefits in suppressing high insulin-induced vascular smooth muscle cell dysfunction have been attributed to its pleiotropic effects on inflammation, apoptosis, and reactive oxygen species (ROS) regulation [34]. Intriguingly, recent evidence suggests that KDM3A can regulate mitochondrial biogenesis and function in other cellular contexts [35]. Nevertheless, whether and how KDM3A participates in the pathogenesis of POI remains unknown.

Based on these observations, we hypothesized that KDM3A protects against POI by preserving mitochondrial function in ovarian granulosa cells through transcriptional activation of key downstream targets. To test this hypothesis, we examined KDM3A expression in clinical POI samples and a cisplatin‑induced POI mouse model. Using KDM3A knockout and ovarian‑specific overexpression approaches, we found that KDM3A is essential for maintaining ovarian function, follicular development, and fertility. Chromatin immunoprecipitation sequencing (ChIP‑seq) identified ATP‑binding cassette sub‑family A member 7 (ABCA7) as a direct transcriptional target of KDM3A, and we further demonstrated that the protective effects of KDM3A against mitochondrial dysfunction and apoptosis are largely mediated through ABCA7 upregulation. Notably, direct overexpression of ABCA7 alone phenocopied the therapeutic benefits of KDM3A in POI mice. Collectively, our findings unveil a previously unrecognized KDM3A‑ABCA7 regulatory axis that safeguards mitochondrial homeostasis, offering potential new therapeutic targets for POI.

## Results

### KDM3A expression is downregulated in POI and correlates with ovarian reserve markers

**Fig. 1:**
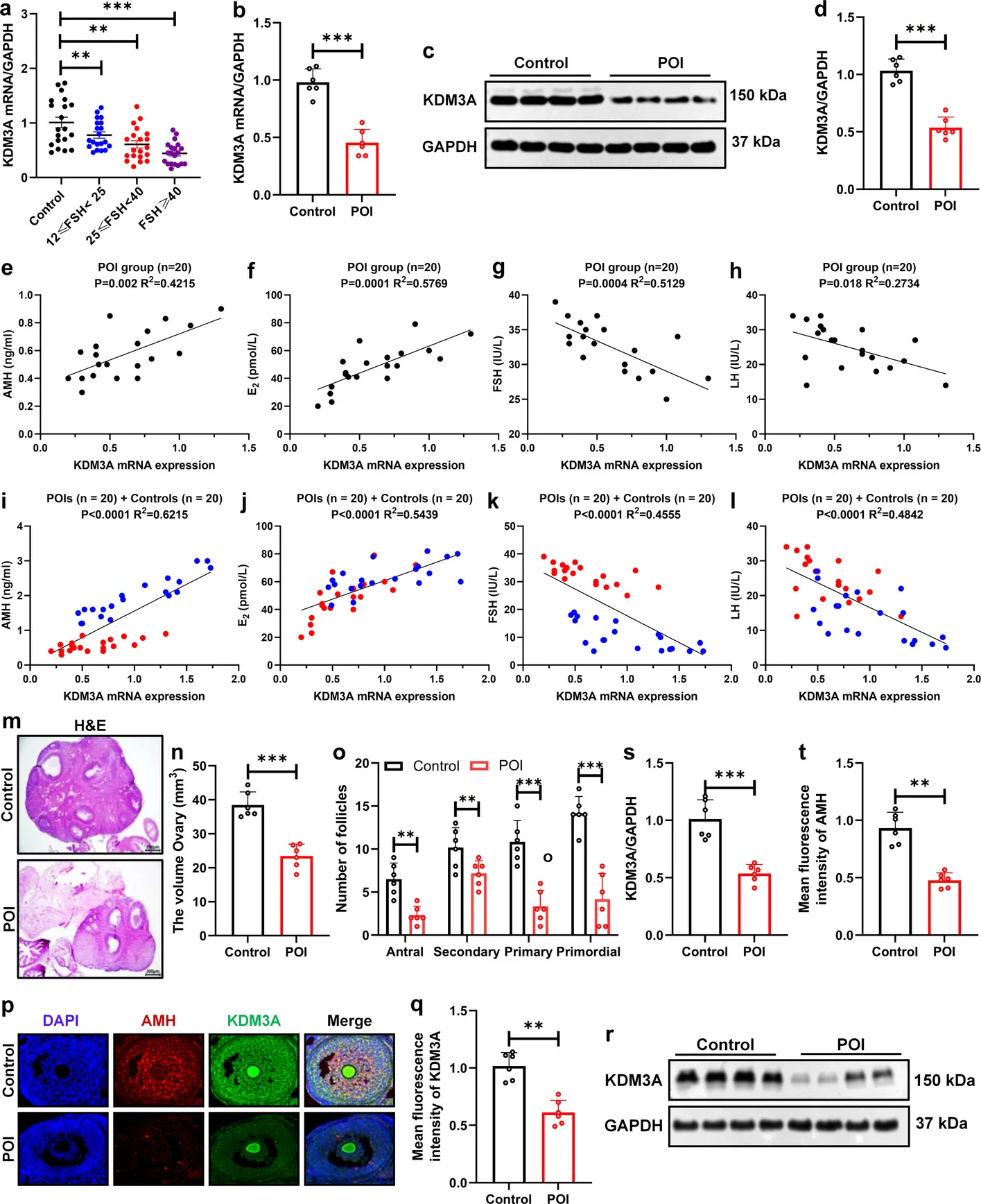
KDM3A expression is downregulated in POI and correlates with ovarian reserve markers. KDM3A protein expression in granulosa cells was assessed by western blot. Correlation analyses were performed between KDM3A mRNA levels in PBMCs and serum hormone concentrations. **a** Relative mRNA expression of *KDM3A* in PBMCs from control women (FSH ≤ 10 IU/L) and patients with varying degrees of elevated FSH levels (12–25 IU/L, 25–40 IU/L, and > 40 IU/L) (*n* = 20 per group). **b-d** *KDM3A* mRNA (**b**) and protein (**c**, **d**) levels in ovarian granulosa cells isolated from healthy control individuals and POI patients (*n* = 6 per group). GAPDH was used as a loading control. **e–h** Pearson correlation analysis between *KDM3A* mRNA expression in PBMCs and serum levels of AMH (**e**), E_2_ (**f**), FSH (**g**), and LH (**h**) in 20 POI patients. **i-l** Pearson correlation analysis between *KDM3A* mRNA expression in PBMCs and serum levels of AMH (**i**), E2 (**j**), FSH (**k**), and LH (**l**) in a combined cohort of POIs (*n* = 20) + Controls (*n* = 20). Control samples are shown in blue dots, and POI samples in red dots. **m** Representative H&E-stained ovarian sections from control and POI model mice. **n** Quantification of ovarian volume. **o** Follicle counts of primordial, primary, secondary, and antral follicles. Immunofluorescence staining (**p**) and quantification (**q**) of KDM3A in ovarian sections. Representative western blots (**r**) and quantification (**s**) of KDM3A protein expression in ovarian tissues. **t** Serum AMH levels measured by ELISA. *P < 0.05, **P < 0.01, ***P < 0.001; ns, not significant. For animal data (**m**–**t**, *n* = 6 per group), statistical comparisons were performed using the Mann–Whitney U test. Data are presented as mean ± SEM”

To investigate KDM3A dysregulation and its correlation with ovarian reserve markers in POI, we measured KDM3A mRNA levels by qRT‑PCR in PBMCs from patients stratified by FSH level and in granulosa cells from a subset of patients. In PBMCs, KDM3A expression was significantly decreased in all patient groups with elevated FSH (12–25 IU/L, 25–40 IU/L, and > 40 IU/L) compared with the control group (FSH ≤ 10 IU/L) (Fig. 1a). Both KDM3A mRNA and protein levels were significantly reduced in granulosa cells from POI patients relative to healthy controls (Fig. 1b-d). In patients with confirmed POI (FSH ≥ 40 IU/L, *n* = 20), KDM3A mRNA levels in PBMCs showed a significant positive correlation with serum AMH and E₂ levels (Fig. 1e, f), and a negative correlation with FSH and LH levels (Fig. 1g, h). These correlative trends were further supported by analysis of the combined cohort, which included POI patients (*n* = 20) and controls (*n* = 20) (Fig. 1i-l). In a cisplatin‑induced POI mouse model, POI mice exhibited significantly reduced ovarian volume (Fig. 1m, n) and a marked depletion of follicles at all developmental stages (Fig. 1o). Immunofluorescence staining revealed that KDM3A protein expression was substantially decreased in ovarian sections from POI mice (Fig. 1p, q), and this downregulation was further confirmed by western blot analysis (Fig. 1r, s). In addition, serum AMH levels were significantly lower in POI model mice compared with controls (Fig. 1t).Collectively, these data indicate that reduced KDM3A expression is associated with POI in both humans and mice and correlates with key ovarian reserve markers. However, given the heterogeneity of POI etiologies, further studies in diverse patient populations are warranted to evaluate the generalizability of KDM3A as a biomarker.

### KDM3A knockout and ovarian‑specific overexpression reveal its protective role in POI

**Fig. 2:**
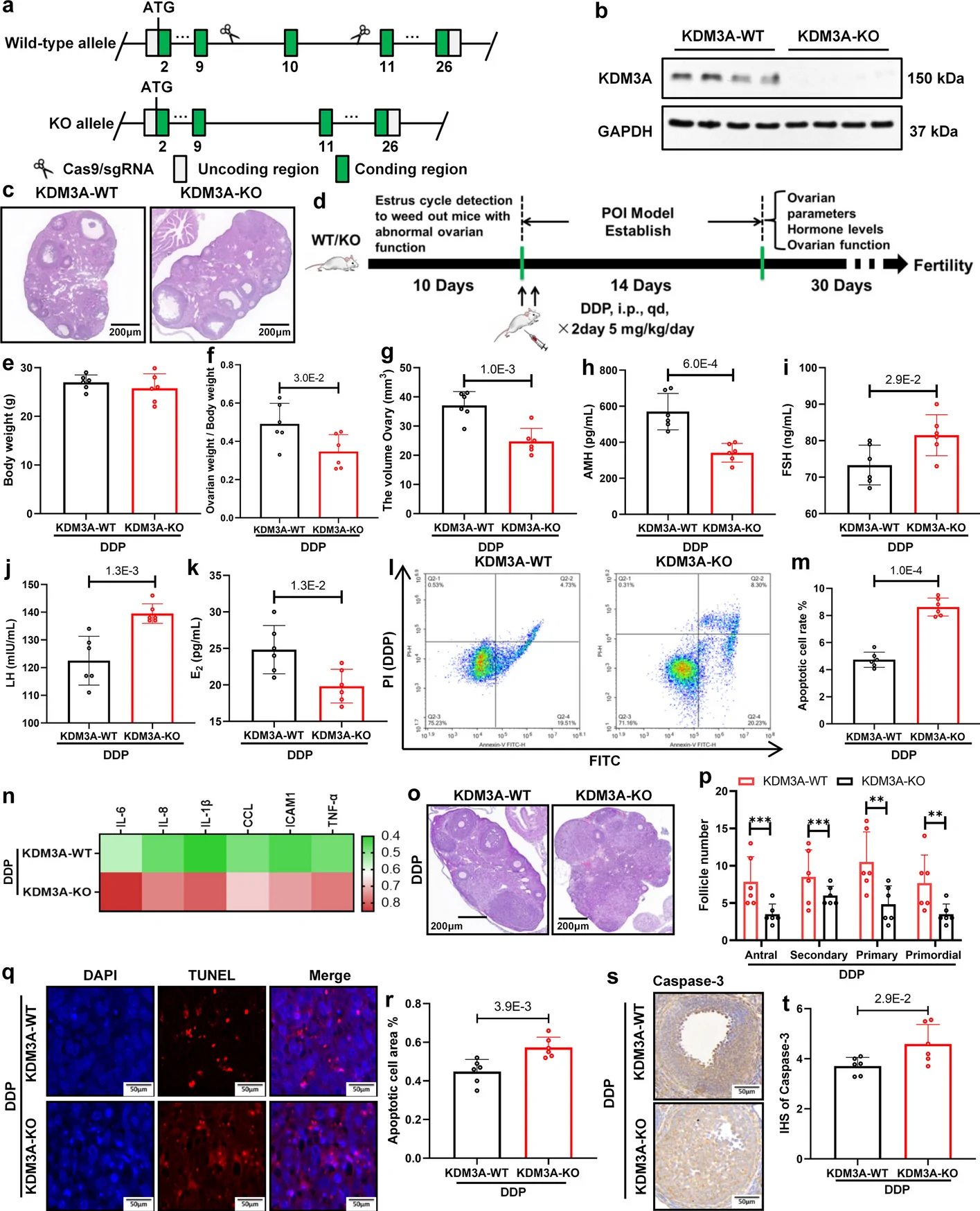
KDM3A deficiency exacerbates ovarian dysfunction in a cisplatin-induced POI mouse model.** a** Schematic diagram of the KDM3A knockout strategy using CRISPR/Cas9 technology. **b** Genotyping PCR analysis confirming the knockout of *Kdm3a* in KDM3A⁻/⁻ mice compared to wild-type (KDM3A⁺/⁺) littermates. **c** Representative H&E-stained images of ovarian sections from KDM3A⁺/⁺ and KDM3A⁻/⁻ mice under basal conditions. Scale bar, 200 μm. **d** Experimental timeline for cisplatin (DDP)-induced POI model establishment and analysis. **e–g** Body weight change (**e**), ovarian index (ovary weight/body weight) (**f**), and ovarian volume (**g**) of KDM3A⁺/⁺ and KDM3A⁻/⁻ mice after cisplatin treatment (*n* = 6 mice per group). **h–k** Serum hormone levels of AMH (**h**), FSH (**i**), LH (**j**), and estradiol (E_2_) (**k**) in the indicated mouse groups after cisplatin treatment (*n* = 6 mice per group). **l**,** m** Flow cytometry analysis of apoptosis in isolated ovarian granulosa cells using Annexin V/PI staining. **l** Representative flow plots. **m** Quantification of apoptotic cell rates (*n* = 6 mice per group). **n** mRNA expression levels of senescence-associated secretory phenotype (SASP) markers in ovarian tissues from the indicated groups (*n* = 6 mice per group). **o**,** p** Ovarian follicle counting. **o** Representative H&E-stained ovarian sections. Scale bar, 200 μm. **p** Quantification of primordial, primary, secondary, and antral follicles (*n* = 6 mice per group, 3 sections per mouse). **q**, **r** TUNEL staining of ovarian sections. **q** Representative images. Red fluorescence indicates TUNEL-positive apoptotic cells. Scale bar, 50 μm. **r** Quantification of TUNEL-positive cells per ovarian section (*n* = 6 mice per group). **s**,**t** Immunohistochemical staining for Cleaved Caspase-3 in ovarian tissues. **s** Representative images. Brown staining indicates positive cells. Scale bar, 50 μm. **t** Quantification of Cleaved Caspase-3-positive cells per ovarian section (*n* = 6 mice per group). *P < 0.05, **P < 0.01, ***P < 0.001; ns, not significant. Data are presented as mean ± SEM. Statistical comparisons between two groups were performed using the Mann–Whitney U test, and comparisons among multiple groups were performed using the Kruskal–Wallis test followed by Dunn’s post-hoc test

To determine whether KDM3A plays a causal role in POI, we generated KDM3A knockout (KDM3A⁻/⁻) mice using CRISPR/Cas9 (Fig. 2a), and confirmed knockout efficiency by genotyping (Fig. 2b). Under untreated conditions, KDM3A⁻/⁻ mice showed no significant differences in ovarian morphology (Fig. 2c), ovarian index, ovarian volume, serum hormone levels (AMH, FSH, LH, E₂), or follicle counts compared with KDM3A⁺/⁺ littermates (Fig. S1), indicating that KDM3A deletion alone does not impair baseline ovarian function. After cisplatin treatment (Fig. 2d), body weight was comparable between the two groups (Fig. 2e), excluding systemic toxicity as a confounder. However, KDM3A⁻/⁻ mice exhibited significantly lower ovarian index and ovarian volume (Fig. 2f, g), elevated serum FSH and LH and reduced AMH and E₂ (Fig. 2h-k), higher granulosa cell apoptosis (Fig. 2l, m), increased SASP marker expression (Fig. 2n), and reduced follicle counts at all stages (Fig. 2o, p). TUNEL staining and cleaved caspase‑3 expression were also significantly elevated in the ovaries of KDM3A⁻/⁻ mice (Fig. 2q-t).

**Fig. 3:**
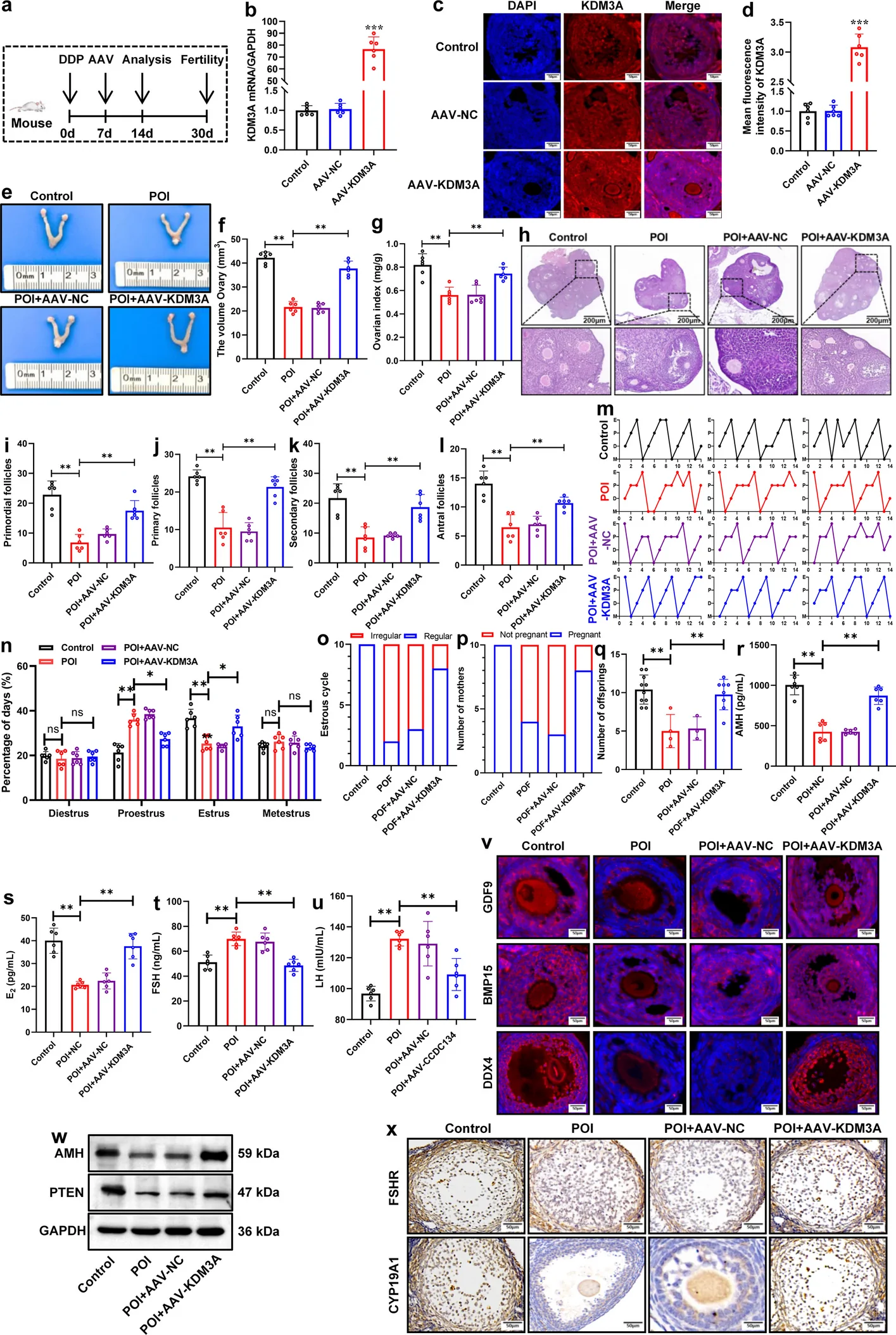
Ovarian-specific overexpression of KDM3A rescues cisplatin-induced ovarian damage, dysfunction, and fertility in POI mice. **a** Experimental design for AAV-mediated ovarian‑specific KDM3A overexpression in the cisplatin‑induced POI mouse model. **b–d** Validation of KDM3A overexpression in ovarian tissues. **b** Relative Kdm3a mRNA levels measured by qRT‑PCR. **c** Representative immunofluorescence images of KDM3A (scale bar, 50 μm). **d** Quantitative analysis of KDM3A fluorescence intensity. **e–g** Ovarian volume and organ coefficient. **e** Representative ovarian morphology. **f** Quantification of ovary volume. **g** Ovarian organ coefficient. **h**–**l** Histological analysis of follicular development. **h** Representative H&E‑stained ovarian sections (scale bar, 200 μm). **i–l** Quantification of follicles at different stages: primordial (**i**), primary (**j**), secondary (**k**), and antral (**l**) follicles. **m–o** Estrous cycle analysis. **m** Representative daily vaginal cytology profiles. **n** Distribution of estrous cycle stages. **o** Percentage of mice with normal estrous cycles. **p**,** q** Fertility assessment. **p** Percentage of productive female mice after mating. **q** Number of pups per litter. **r–u** Serum hormone levels measured by ELISA: **r**, AMH; **s**, E₂; **t** FSH; **u**, LH; **v**, Immunofluorescence analysis of oocyte‑specific markers (GDF9, BMP15, DDX4) (scale bar, 50 μm). **w** Western blot analysis of granulosa cell markers (AMH and PTEN). **x** Immunohistochemical staining of steroidogenic regulators (FSHR and CYP19A1) in granulosa cells (scale bar, 50 μm). Data are presented as mean ± SEM (*n* = 6 mice per group). *P < 0.05, **P < 0.01, ***P < 0.001; ns, not significant. Statistical comparisons between two groups were performed using the Mann–Whitney U test, and comparisons among multiple groups were performed using the Kruskal–Wallis test followed by Dunn’s post‑hoc test

To test whether KDM3A restoration could rescue ovarian function, we employed AAV‑mediated ovarian‑specific KDM3A overexpression in cisplatin‑induced POI mice (Fig. 3a). The POI + AAV‑KDM3A group showed significantly elevated KDM3A mRNA levels (Fig. 3b) and immunofluorescence intensity (Fig. 3c, d) compared with POI and POI + AAV‑NC groups, along with increased ovarian organ coefficient (Fig. 3e-g). Histological analysis revealed markedly restored follicular reserve, with significantly higher numbers of primordial, primary, secondary, and antral follicles (Fig. 3h-l). Functional assessment showed that AAV‑KDM3A treatment significantly improved estrous cycle regularity (Fig. 3m-o), increased both litter size and the percentage of productive females (Fig. 3p-q), and restored serum AMH, E₂, FSH, and LH levels to values comparable to normal controls (Fig. 3r-u). Immunofluorescence confirmed restoration of oocyte‑specific markers GDF9, BMP15, and DDX4 (Fig. 3v); western blotting confirmed recovery of granulosa cell markers AMH and PTEN (Fig. 3w); and immunohistochemistry demonstrated upregulation of steroidogenic regulators FSHR and CYP19A1 (Fig. 3x). Collectively, these findings indicate that KDM3A deficiency does not affect ovarian function under physiological conditions but significantly exacerbates cisplatin‑induced injury, whereas ovarian‑specific KDM3A overexpression effectively rescues ovarian morphology, function, and fertility.

### KDM3A overexpression suppresses apoptosis and maintains mitochondrial function in granulosa cells

**Fig. 4:**
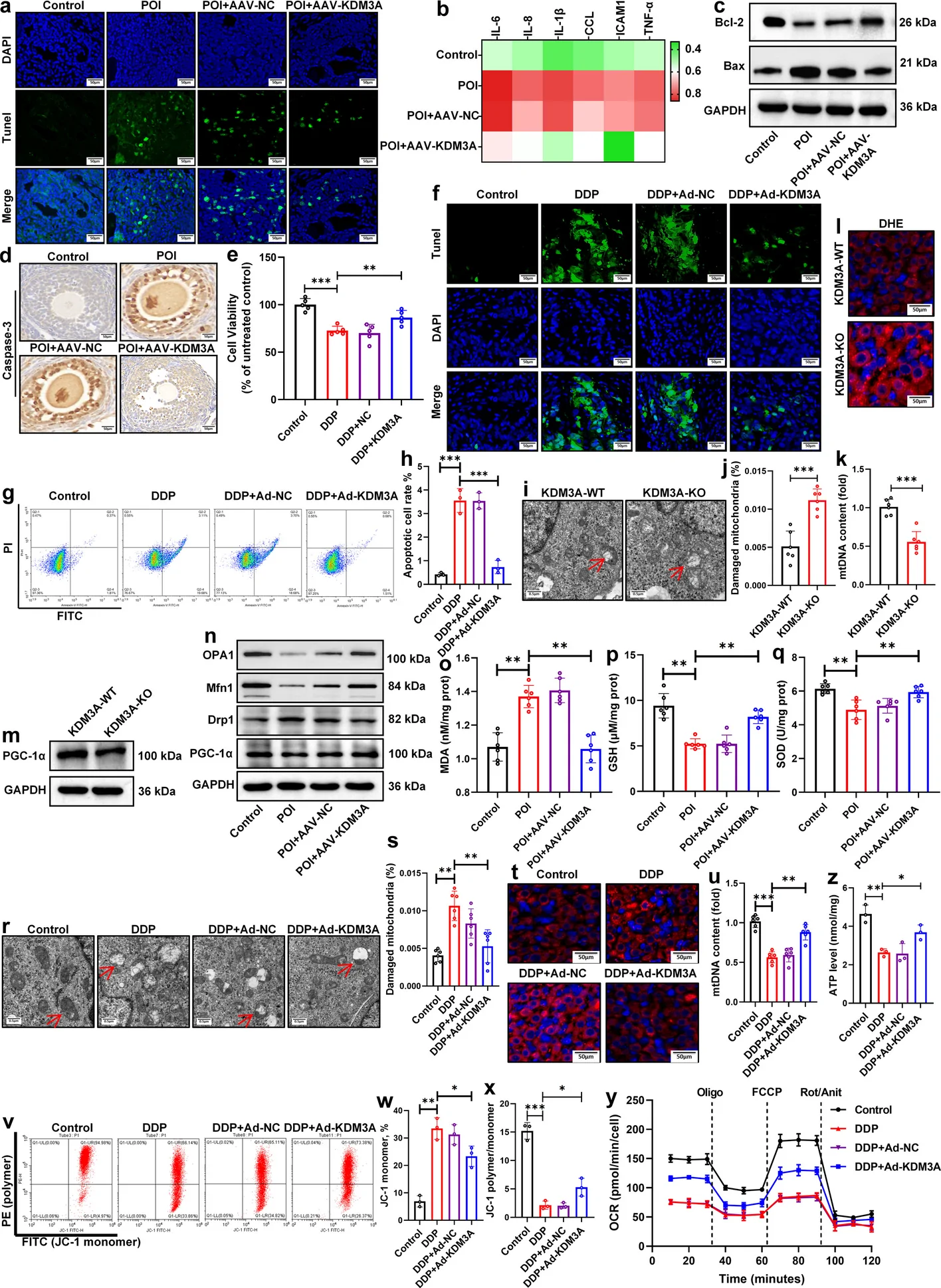
KDM3A overexpression suppresses apoptosis and preserves mitochondrial homeostasis in granulosa cells in vivo and in vitro. **a** TUNEL staining of apoptotic cells in ovarian sections from control, POI, and POI + AAV‑KDM3A mice. **b** mRNA expression of SASP markers in ovarian tissues. **c** Western blot analysis of Bax and Bcl‑2 in ovarian tissues with quantification. **d** Immunohistochemical staining and quantification of cleaved caspase‑3 in ovarian sections. **e** Cell viability of KGN cells treated with cisplatin (5 μg/mL, 24 h) with or without Ad‑KDM3A. **f–h** Apoptosis in KGN cells assessed by TUNEL staining (**f**) and flow cytometry (Annexin V/PI, **g** and **h**). **i**,** j** TEM of granulosa cells from KDM3A⁺/⁺ and KDM3A⁻/⁻ mice showing mitochondrial ultrastructure (**i**) and damage scoring (**j**). **k** Relative mtDNA copy number. **l** Intracellular ROS levels by DCFH‑DA staining. **m** Western blot of PGC‑1α expression. **n** Western blot analysis of mitochondrial dynamics regulators (OPA1, Mfn1, Drp1) and PGC‑1α in ovarian tissues from control, POI, POI + AAV‑NC, and POI + AAV‑KDM3A mice, with quantification. **o–q** Oxidative stress parameters: MDA (**o**), GSH (**p**), and SOD activity (**q**). **r**,** s** TEM of KGN cells treated with cisplatin ± Ad‑KDM3A, showing images (**r**) and damage scoring (**s**). **t** ROS levels. **u** mtDNA copy number. **v–x** ΔΨm by JC‑1 staining: representative flow plots (**v**), monomer fluorescence (**w**), and polymer/monomer ratio (**x**). **y**,** z** Oxygen consumption rate (OCR, **y**) and ATP levels (**z**). Data are presented as mean ± SEM. For in vivo experiments (**a**–**d**, **i**–**q**), *n* = 6 mice per group; for in vitro experiments (**e**–**h**, **r**–**z**), *n* = 3 independent experiments. *P < 0.05, **P < 0.01, ***P < 0.001; ns, not significant. Animal data were analyzed using Mann–Whitney U test (two groups) or Kruskal–Wallis with Dunn’s post‑hoc test (multiple groups); in vitro data were analyzed using unpaired t‑test or one‑way ANOVA with Tukey’s post‑hoc test as appropriate

To investigate whether KDM3A protects granulosa cells by inhibiting apoptosis and preserving mitochondrial homeostasis, we evaluated these processes in vivo and in vitro. In vivo, TUNEL staining showed that the elevated apoptosis rate in POI ovaries was markedly attenuated by AAV‑KDM3A treatment (Fig. 4a), and SASP marker expression was also significantly reduced (Fig. 4b). Western blot analysis revealed that cisplatin increased the pro‑apoptotic protein Bax and decreased the anti‑apoptotic protein Bcl‑2, effects reversed by AAV‑KDM3A (Fig. 4c), and immunohistochemistry confirmed reduced cleaved caspase‑3 levels following KDM3A overexpression (Fig. 4d). In vitro, KDM3A overexpression in KGN cells was confirmed by increased mRNA and protein levels (Fig. S2a, b). Cisplatin reduced KGN cell viability in a dose‑ and time‑dependent manner (Fig. S2c, d). At 5 μg/mL for 24 h, KDM3A overexpression significantly rescued cell viability (Fig. 4e) and reduced apoptosis by TUNEL and flow cytometry (Fig. 4f-h).

Under basal conditions, TEM revealed normal mitochondrial morphology in KDM3A⁺/⁺ granulosa cells, whereas KDM3A⁻/⁻ cells exhibited severe structural damage including cristae disorganization, matrix granule loss, and vacuolization (Fig. 4i-j). KDM3A⁻/⁻ cells also showed reduced mtDNA copy number (Fig. 4k), elevated ROS (Fig. 4l), and decreased PGC‑1α expression (Fig. 4m), indicating impaired mitochondrial biogenesis. In POI mice, ovarian tissues showed reduced OPA1 (a mitochondrial inner membrane fusion protein), Mfn1 (a mitochondrial outer membrane fusion protein), and PGC‑1α, with elevated Drp1 (a mitochondrial fission protein); AAV‑KDM3A reversed these abnormalities (Fig. 4n). Oxidative stress markers (MDA, SOD, GSH) were similarly ameliorated (Fig. 4o-q). In vitro, cisplatin‑induced mitochondrial damage in KGN cells—including swelling, outer membrane rupture, and cristae disorganization—was markedly ameliorated by Ad‑KDM3A (Fig. 4r, s), which also reduced ROS (Fig. 4t), restored mtDNA copy number (Fig. 4u), reversed JC‑1 depolarization (Fig. 4v-x), and improved both OCR and ATP generation (Fig. 4y, z). These findings indicate that KDM3A overexpression protects granulosa cells from cisplatin‑induced apoptosis and mitochondrial dysfunction by preserving mitochondrial structure, reducing oxidative stress, and maintaining bioenergetic capacity.

### KDM3A regulates mitochondrial homeostasis by targeting the ABCA7 promoter region

**Fig. 5:**
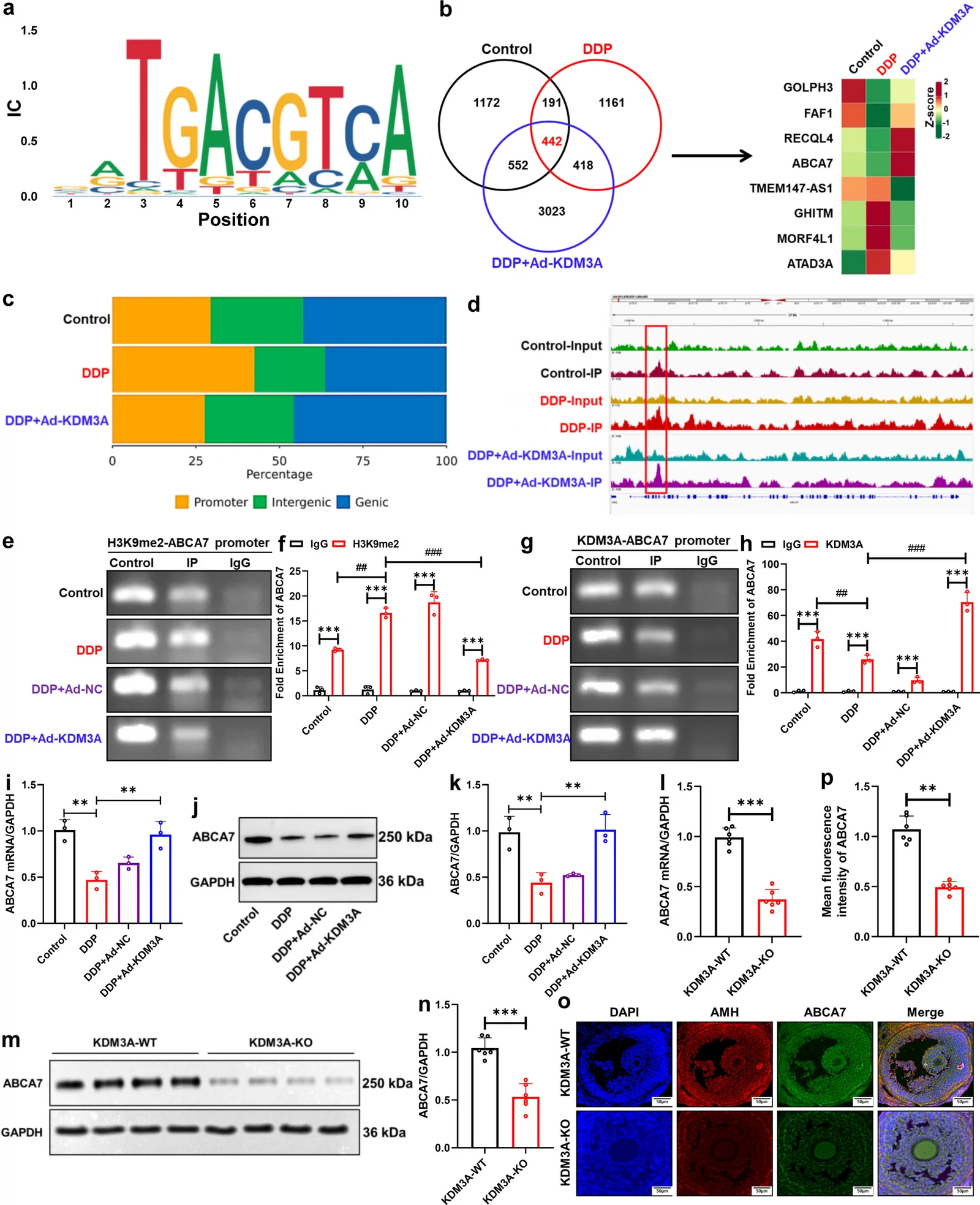
KDM3A transcriptionally regulates ABCA7 expression by binding to its promoter region. **a** De novo motif analysis from ChIP-seq data showing the characteristic binding motif of KDM3A in granulosa cells. **b** Venn diagram displaying the overlap of KDM3A binding sites identified in granulosa cells from Control, DDP, and DDP + Ad-KDM3A groups (*n* = 3 independent experiments). **c** Genomic distribution of KDM3A binding peaks from ChIP-seq analysis, showing enrichment in promoter regions. **d** Browser tracks of ChIP-seq signals at the ABCA7 promoter region in the three experimental groups. **e**, **f** ChIP-qPCR analysis of H3K9me2 enrichment at the ABCA7 promoter. **e** Schematic of the ABCA7 promoter region showing the amplified segment. **f** Quantification of H3K9me2 binding (*n* = 3 independent experiments). **g**, **h** ChIP-qPCR analysis of KDM3A enrichment at the ABCA7 promoter. **g** Schematic of the amplified region. **h** Quantification of KDM3A binding (*n* = 3 independent experiments). **i-k** Effect of KDM3A overexpression on ABCA7 expression in granulosa cells. **i** ABCA7 mRNA levels by qRT-PCR. **j**,** k** Representative western blot (**j**) and quantification (**k**) of ABCA7 protein levels (*n* = 3 independent experiments). **l-p** ABCA7 expression in KDM3A knockout ovaries. **l** ABCA7 mRNA levels by qRT-PCR. **m–o** Representative western blot (**m**) and quantification of ABCA7 protein levels in KDM3A⁺/⁺ and KDM3A⁻/⁻ mice (**n**, **o**) (*n* = 6 mice/group). *P < 0.05, **P < 0.01, ***P < 0.001; ns, not significant. For animal data (**l**–**o**, *n* = 6 per group), statistical comparisons were performed using the Mann–Whitney U test. Data are presented as mean ± SEM. For in vitro experiments (**i**–**r**, *n* = 3 independent experiments), statistical comparisons were performed using unpaired t-test or one-way ANOVA with Tukey’s post-hoc test as appropriate

To identify direct transcriptional targets of KDM3A, we performed ChIP‑seq on granulosa cells from Control, cisplatin (DDP), and DDP + Ad‑KDM3A groups, followed by de novo motif analysis, peak distribution analysis, and cross‑referencing with the MitoCarta3.0 database. ChIP‑qPCR was used to validate KDM3A and H3K9me2 binding to the ABCA7 promoter. De novo motif analysis revealed characteristic KDM3A binding sequences, validating the ChIP‑seq data (Fig. 5a). Intersection analysis identified 442 common KDM3A binding events across the three groups (Fig. 5b), with dynamic distribution across genomic regions and increased promoter enrichment under cisplatin‑induced stress (Fig. 5c). Cross‑referencing with MitoCarta3.0 identified ABCA7 as a potential downstream target, with KDM3A binding peaks confirmed at the ABCA7 promoter in all three groups (Fig. 5d). ChIP‑qPCR showed that H3K9me2 enrichment at the ABCA7 promoter was significantly higher in DDP and DDP + Ad‑NC groups compared with controls, whereas DDP + Ad‑KDM3A significantly reduced H3K9me2 binding (Fig. 5e, f). Conversely, KDM3A binding was significantly reduced in DDP and DDP + Ad‑NC groups but increased following Ad‑KDM3A treatment (Fig. 5g, h). In DDP‑treated cells, ABCA7 mRNA and protein were significantly reduced, whereas KDM3A overexpression elevated ABCA7 expression (Fig. 5i-k). Consistently, ABCA7 expression was reduced in KDM3A⁻/⁻ ovaries (Fig. 5l-o). Collectively, these findings indicate that KDM3A directly binds to the ABCA7 promoter, reduces H3K9me2 occupancy, and activates ABCA7 transcription.

### ABCA7 overexpression rescues ovarian dysfunction by suppressing apoptosis and maintaining mitochondrial function

**Fig. 6:**
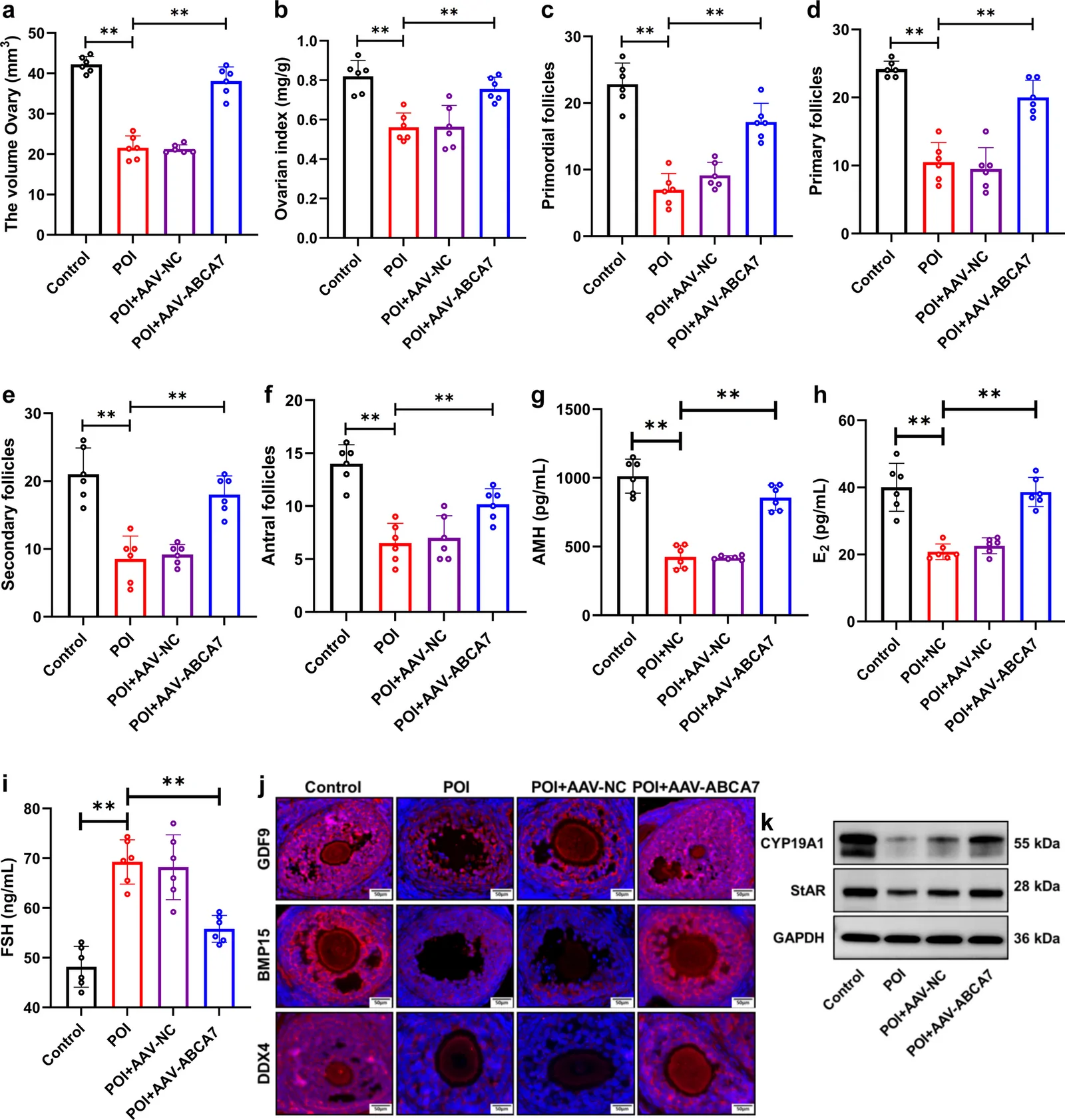
ABCA7 overexpression rescues ovarian function in cisplatin-induced POI mice. **a** Ovarian volume measurement in control, POI, POI + AAV-NC, and POI + AAV-ABCA7 groups. **b** Ovarian organ coefficient (ovary weight/body weight) (*n* = 6 mice/group). **c-f** Quantification of follicular development: primordial follicles (**c**), primary follicles (**d**), secondary follicles (**e**), and antral follicles (**f**) (*n* = 6 mice/group). **g-i** Serum hormone levels. **g** AMH levels. **h** E_2_ levels. **i** FSH levels measured by ELISA (*n* = 6 mice/group). **j** Expression of oocyte-specific markers (*n* = 6 mice/group). **k** Expression of steroidogenic regulators (*n* = 6 mice/group). *P < 0.05, **P < 0.01, ***P < 0.001; ns, not significant. Data are presented as mean ± SEM. Statistical comparisons among multiple groups were performed using the Kruskal–Wallis test followed by Dunn’s post-hoc test

To determine whether ABCA7 is sufficient to rescue ovarian dysfunction in POI, we overexpressed ABCA7 in cisplatin‑induced POI mice using AAV‑ABCA7. AAV‑ABCA7 treatment significantly increased ABCA7 mRNA and protein levels in ovarian tissues (Fig. S3).Compared with POI and POI + AAV‑NC groups, AAV‑ABCA7‑treated mice showed significantly increased ovarian volume and organ coefficient (Fig. 6a, b), higher follicle counts at all stages (Fig. 6c-f), corrected AMH, E₂, and FSH levels (Fig. 6g-i), restored oocyte‑specific markers GDF9, BMP15, and DDX4 (Fig. 6j), and upregulated steroidogenic regulators StAR and CYP19A1 (Fig. 6k).

**Fig. 7:**
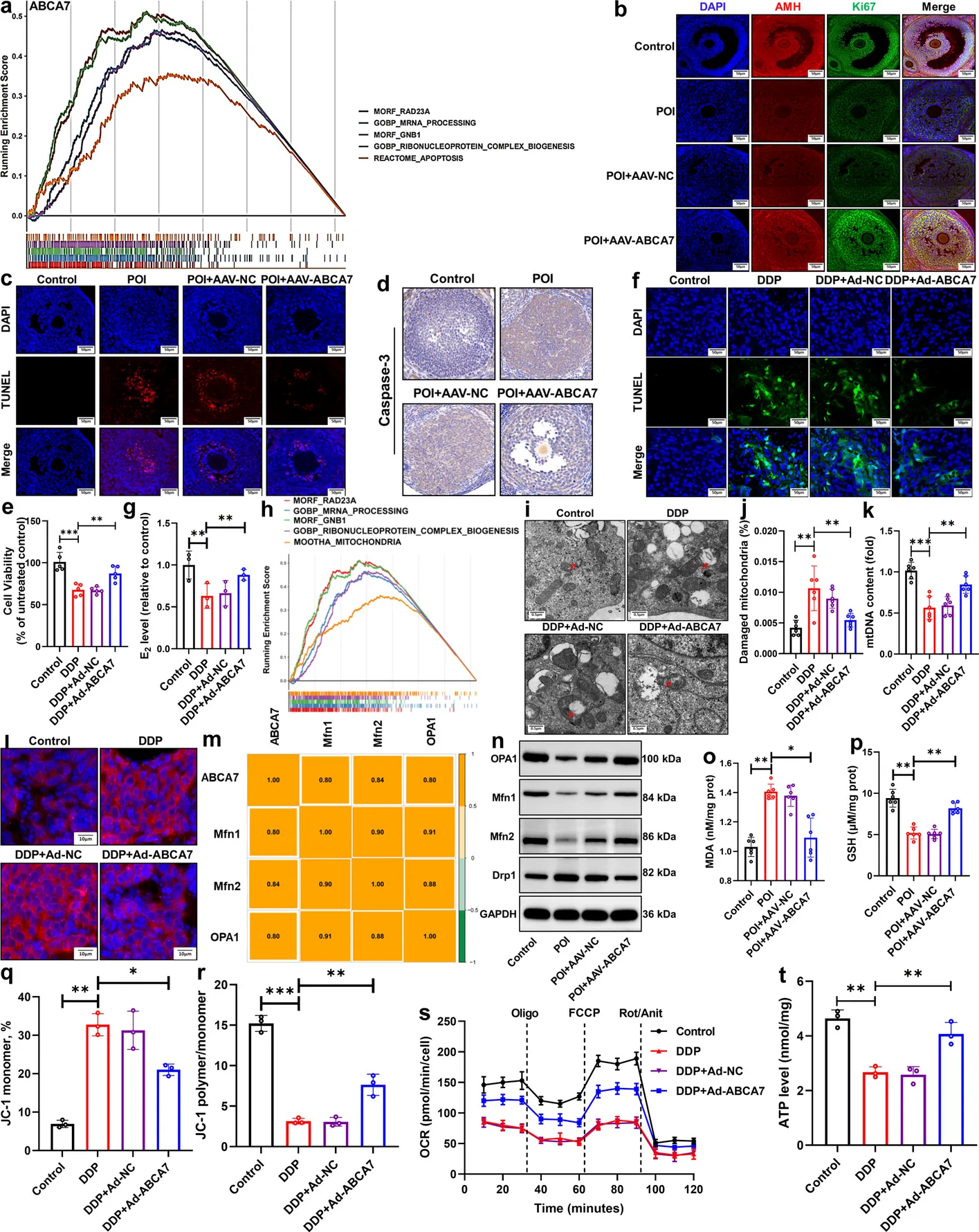
ABCA7 overexpression rescues ovarian dysfunction by suppressing apoptosis and maintaining mitochondrial function in POI mice. **a** GSEA plot showing enrichment of the apoptosis pathway with ABCA7 expression. **b** Immunofluorescence staining and quantification of Ki67 in ovarian sections (scale bar, 50 μm). **c** TUNEL staining and quantification of apoptotic cells in ovarian sections. **d** Immunohistochemical staining and quantification of cleaved caspase‑3. **e** Cell viability of KGN cells treated with cisplatin ± Ad‑ABCA7. **f** TUNEL assay in KGN cells with quantification. **g** E₂ concentration in culture supernatants measured by ELISA. **h** GSEA plot showing enrichment of mitochondrial pathways with ABCA7 expression. **i**,** j** Transmission electron microscopy of KGN cells treated with cisplatin ± Ad‑ABCA7, showing representative images (**i**, scale bar 500 nm) and mitochondrial damage scoring (**j**). **k** Relative mtDNA copy number in KGN cells. **l** Intracellular ROS levels detected by DCFH‑DA staining (representative images and quantification, scale bar 50 μm). **m** Correlation analysis between ABCA7 expression and mitochondrial fusion proteins (Mfn1, Mfn2, OPA1). **n** Western blot analysis of OPA1, Mfn1, Mfn2, and Drp1 in ovarian tissues from control, POI, POI + AAV‑NC, and POI + AAV‑ABCA7 mice, with quantification. **o**,** p** Oxidative stress parameters in ovarian tissues: MDA (**o**) and GSH (**p**) levels. **q**,** r** Mitochondrial membrane potential (ΔΨm) assessed by JC‑1 staining, showing representative flow plots (**q**) and JC‑1 polymer/monomer ratio (**r**). **s**,** t**, Mitochondrial bioenergetic function: oxygen consumption rate (OCR, **s**) and cellular ATP levels (**t**). Data are presented as mean ± SEM. For in vivo experiments (**a**–**d**, **m**–**p**), *n* = 6 mice per group; for in vitro experiments (**e**–**l**, **q**–**t**), *n* = 3 independent experiments. *P < 0.05, **P < 0.01, ***P < 0.001; ns, not significant. Animal data were analyzed using Mann–Whitney U test (two groups) or Kruskal–Wallis with Dunn’s post‑hoc test (multiple groups); in vitro data were analyzed using unpaired t‑test or one‑way ANOVA with Tukey’s post‑hoc test as appropriate

GSEA revealed significant enrichment of ABCA7 in the apoptosis pathway (Fig. 7a). In vivo, AAV‑ABCA7 increased Ki67 expression (Fig. 7b), reduced TUNEL‑positive cells (Fig. 7c), and decreased cleaved caspase‑3 (Fig. 7d). In vitro, ABCA7 overexpression in KGN cells improved cell viability after cisplatin treatment (Fig. 7e), reduced apoptosis by TUNEL (Fig. 7f), and restored E₂ in culture supernatants (Fig. 7g). GSEA also showed ABCA7 association with the mitochondrial pathway (Fig. 7h). To further explore this, we examined mitochondrial morphology and function. In vitro, Ad‑ABCA7 ameliorated cisplatin‑induced mitochondrial damage (Fig. 7i, j), restored mtDNA copy number (Fig. 7k), and reduced ROS levels (Fig. 7l). Correlation analysis revealed strong positive correlations between ABCA7 and mitochondrial fusion proteins Mfn1, Mfn2, and OPA1 (Fig. 7m). In vivo, POI ovaries showed reduced OPA1, Mfn1, and Mfn2 with elevated Drp1; AAV‑ABCA7 restored fusion proteins and reduced Drp1 (Fig. 7n), and ameliorated oxidative stress markers MDA and GSH (Fig. 7o, p). Furthermore, Ad‑ABCA7 reversed JC‑1 depolarization (Fig. 7q, r), and improved OCR and ATP generation (Fig. 7s, t). These findings indicate that ABCA7 overexpression rescues ovarian dysfunction in POI mice by suppressing apoptosis and preserving mitochondrial structure and function, supporting ABCA7 as a key mediator of KDM3A's protective effects.

## Discussion

Epigenetic dysregulation has emerged as a key factor in the pathogenesis of POI [36, 37]. While previous studies have implicated various histone modifiers in ovarian function, the role of histone demethylation—particularly H3K9me2 demethylation—remains poorly understood. In this study, we identified KDM3A, a H3K9me2 demethylase, as a critical protective factor in POI. Our findings indicate that KDM3A is downregulated in both idiopathic POI patients and a cisplatin-induced mouse model, extending the known roles of KDM3A beyond metabolic and vascular diseases into reproductive pathology [31, 38]. These results position KDM3A as a novel epigenetic regulator in ovarian homeostasis.

The correlation between KDM3A expression and ovarian reserve markers (AMH, FSH, LH, E_2_) in our patient cohort suggests that KDM3A may serve as a potential indicator of ovarian function. This is consistent with emerging evidence linking epigenetic regulators to ovarian aging [39, 40]. However, unlike genetic mutations that directly cause follicular depletion, KDM3A downregulation appears to reflect a stress-responsive epigenetic change. The observation that KDM3A is already reduced in patients with incipient ovarian insufficiency (FSH 12–25 IU/L) suggests that epigenetic alterations may precede clinically detectable hormonal changes. This temporal relationship warrants further investigation in longitudinal studies.

Our loss- and gain-of-function experiments establish a causal role for KDM3A in protecting against chemotherapy-induced ovarian injury. While KDM3A deficiency alone did not impair baseline ovarian function, it significantly exacerbated cisplatin-induced damage, indicating that KDM3A functions primarily as a stress-responsive protector rather than a developmental necessity. This context-dependent role parallels findings in other tissues, where KDM3A is dispensable under basal conditions but becomes critical under metabolic or oxidative stress [14, 41, 42]. The ability of ovarian-specific KDM3A overexpression to rescue follicular depletion and restore fertility highlights its therapeutic potential, particularly for iatrogenic POI in cancer survivors.

Mitochondrial dysfunction is increasingly recognized as a central driver of POI pathogenesis [43–45]. Our study provides the first evidence linking KDM3A to mitochondrial regulation in ovarian cells. KDM3A-deficient granulosa cells exhibited hallmark features of mitochondrial dysfunction—ROS accumulation, loss of membrane potential, and reduced ATP production—consistent with phenotypes observed in KDM3A-related metabolic disorders [46]. Notably, we found that KDM3A overexpression restored mitochondrial dynamics by upregulating fusion proteins (OPA1, Mfn1/2) and suppressing the fission protein Drp1. This dual regulation of fusion-fission balance aligns with reports in other cell types, where KDM3A influences mitochondrial morphology through transcriptional control of dynamic regulators [47–51]. However, the precise mechanism by which KDM3A coordinates these changes in granulosa cells—whether directly or via downstream effectors—requires further investigation.

A key finding of this study is the identification of ABCA7 as a direct transcriptional target of KDM3A. ABCA7 is best known for its role in lipid transport and has been extensively studied in the context of Alzheimer's disease [52]. Kawatani et al. demonstrated that ABCA7 deficiency in human iPSC-derived neurons leads to compromised mitochondrial respiration, excessive ROS generation, and altered mitochondrial morphology [53]. Similarly, a recent study reported that ABCA7 loss-of-function variants disrupt mitochondrial function and phosphatidylcholine metabolism in excitatory neurons [54], and neuron-specific Abca7 knockout mice exhibited impaired mitochondrial homeostasis and reduced synaptic protein levels [55]. Our findings extend this emerging paradigm to ovarian granulosa cells, demonstrating that ABCA7 mediates KDM3A's protective effects on mitochondrial morphology, membrane potential, and ATP production. The observation that ABCA7 overexpression alone rescues the POI phenotype suggests that ABCA7 is not merely a passive downstream marker but an active functional mediator. This suggests that ABCA7 may represent a potential therapeutic target for conditions involving mitochondrial dysfunction, beyond its established roles in lipid metabolism and neurodegeneration.

The emerging landscape of epigenetic regulation in POI includes diverse players such as FOXJ2 and PCBP2 [41, 56]. While FOXJ2 has been implicated in granulosa cell proliferation and PCBP2 in IGF2-mediated mitochondrial function, our study identifies a distinct mechanism—histone demethylation—as a key regulatory layer. The KDM3A-ABCA7 axis is unique in that it directly links nuclear epigenetic status to mitochondrial lipid homeostasis, a connection not previously described in ovarian biology. This distinction is important because it suggests that therapies targeting histone methylation (e.g., demethylase activators) could complement existing approaches aimed at mitochondrial antioxidants or anti-apoptotic factors. Future studies should explore whether KDM3A and other epigenetic regulators converge on common downstream pathways or operate in parallel to maintain ovarian reserve.

While this study focused on granulosa cells, we acknowledge that POI pathogenesis involves complex interactions among multiple ovarian cell types, including oocytes, theca cells, and stromal cells. Granulosa cell dysfunction can indirectly affect oocyte quality and theca cell steroidogenesis through paracrine signaling. Conversely, primary defects in oocytes or theca cells may also influence granulosa cell function. Future studies should investigate whether the KDM3A-ABCA7 axis operates in other ovarian cell types and how cross-talk between these compartments contributes to POI pathogenesis.

Several limitations should be acknowledged. First, although our findings suggest a potential role for KDM3A as a biomarker of ovarian reserve, POI is etiologically heterogeneous, encompassing genetic, autoimmune, iatrogenic, and idiopathic forms. Our study was limited to patients with idiopathic POI, and it remains to be determined whether KDM3A dysregulation occurs in other subtypes. Therefore, careful patient phenotyping is essential in future validation studies. Second, while we identified ABCA7 as a key downstream mediator, the precise molecular mechanism by which ABCA7 regulates mitochondrial function in granulosa cells remains unclear. Given ABCA7's established role as a lipid transporter, it may influence mitochondrial membrane lipid composition, thereby affecting membrane potential and respiratory chain efficiency. Lipidomic analyses will be necessary to test this hypothesis. Third, our findings are derived primarily from a cisplatin-induced POI model, which recapitulates chemotherapy-related ovarian injury but does not fully represent the heterogeneity of human POI, particularly genetic or autoimmune forms with gradual onset. Validation in additional models—such as Fmr1 premutation mice or autoimmune POI models—will be essential to determine the generalizability of the KDM3A-ABCA7 axis. Fourth, although we focused on ABCA7, KDM3A likely regulates a broader transcriptional network. Our ChIP-seq data identified additional mitochondrial-related targets (ATAD3A, GHITM, RECQL4, FAF1, GOLPH3), whose contributions to the POI phenotype warrant further investigation.

Based on these considerations, future studies should: (1) elucidate the lipid-related mechanism of ABCA7 in granulosa cell mitochondria using lipidomic and proteomic approaches; (2) validate the KDM3A-ABCA7 axis in genetic and autoimmune POI models to assess its broader relevance; (3) investigate the functional roles of other KDM3A targets identified in our ChIP-seq analysis; and (4) explore pharmacological strategies to enhance KDM3A activity or deliver ABCA7 specifically to the ovary as potential therapeutic interventions for POI.

**Fig. 8:**
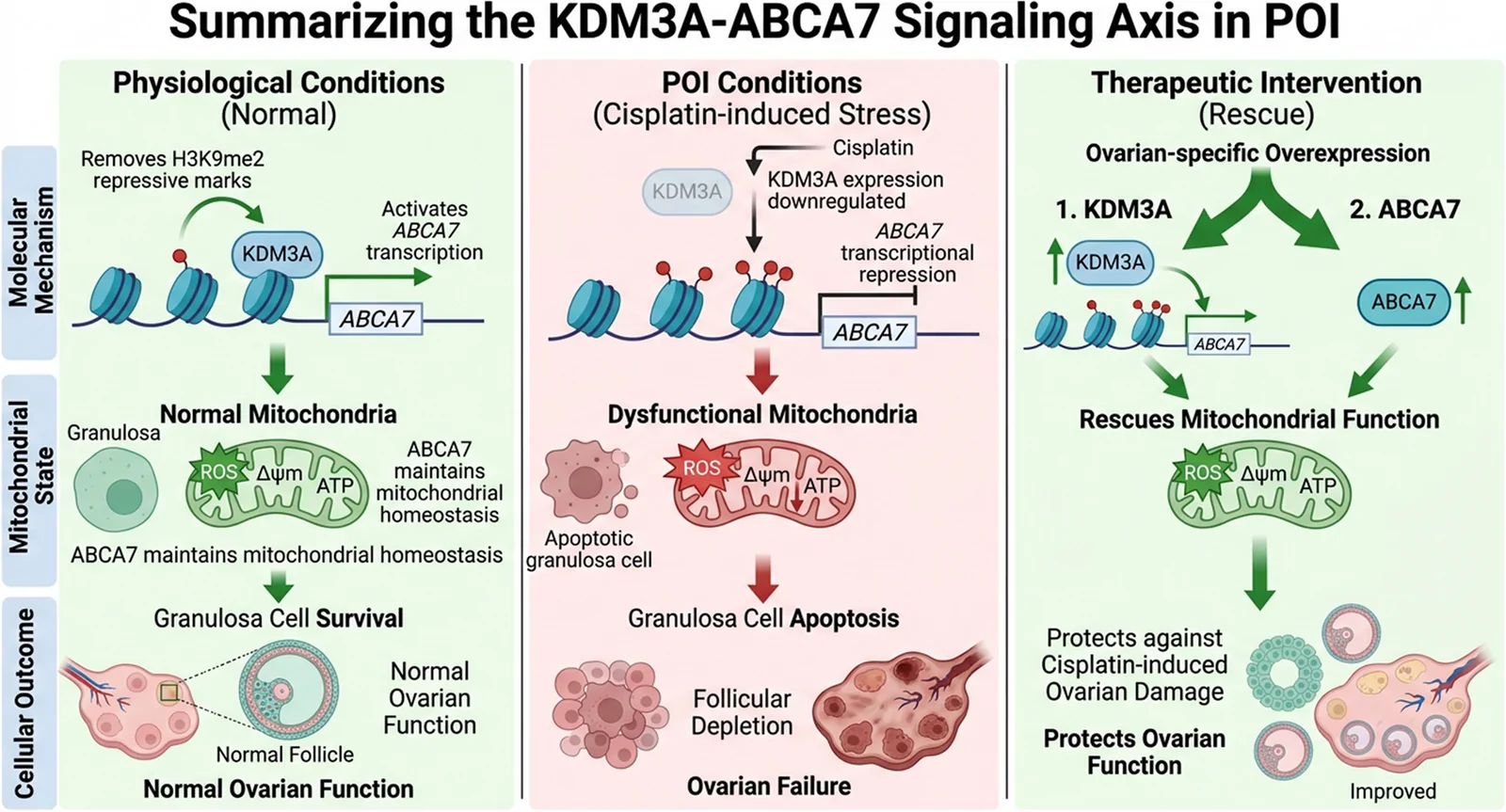
Schematic model of the KDM3A-ABCA7 signaling axis in POI pathogenesis. Under physiological conditions, KDM3A binds to the ABCA7 promoter, demethylates H3K9me2, and activates ABCA7 transcription. ABCA7 maintains mitochondrial homeostasis in granulosa cells, supporting normal follicular development. Under cisplatin-induced stress, KDM3A downregulation leads to increased H3K9me2 enrichment at the ABCA7 promoter and transcriptional repression of ABCA7. This results in mitochondrial dysfunction (ROS accumulation, loss of membrane potential, reduced ATP production), granulosa cell apoptosis, and follicular depletion. Ovarian-specific overexpression of KDM3A or ABCA7 restores mitochondrial function and protects against ovarian injury. Figure 8 was designed and conceptualised entirely by the authors. The visual rendering and layout were assisted by the DroidxAI platform (https://droidxai.com/) as an auxiliary illustration tool. All scientific content, including molecular pathways, cellular components, and mechanistic relationships depicted in the figure, was determined by the authors

In conclusion, this study identifies a KDM3A-ABCA7 epigenetic pathway that protects against cisplatin-induced ovarian dysfunction by maintaining mitochondrial homeostasis in granulosa cells (Fig. 8). These findings provide insights into the mechanisms of chemotherapy-related ovarian injury and suggest that this axis may represent a potential therapeutic target for patients with iatrogenic POI. However, given the etiological heterogeneity of POI, accurate patient phenotyping and validation in diverse disease models will be essential before clinical translation. Further studies are needed to determine whether this pathway plays a broader role in genetic or autoimmune forms of the disease.

## Materials and methods

### Patient populations and peripheral blood collection

POI patients were recruited from the Center for Reproductive Medicine at Shanxi Children's Hospital. Diagnosis followed the 2016 ESHRE guidelines [57, 58], requiring primary or secondary amenorrhea for > 4 months before age 40 and serum FSH > 25 IU/L on two occasions > 4 weeks apart. Patients with known etiologies were excluded based on karyotyping, FMR1 testing, autoimmune screening, history of pelvic surgery/chemotherapy/radiotherapy, or metabolic disorders, ensuring all included cases were idiopathic. A total of 80 participants were stratified into four groups by FSH level: Control (≤ 10 IU/L, *n* = 20), Incipient ovarian insufficiency (12–25 IU/L, *n* = 20), Moderate POI (20–40 IU/L, *n* = 20), and Severe POI (≥ 40 IU/L, *n* = 20). The incipient group was included for exploratory analysis. Serum FSH, AMH, LH, and E₂ were measured by chemiluminescent assay [57]. The study was approved by the Medical Ethics Committee of Shanxi Children's Hospital (IRB-GKY-2020–008), and written informed consent was obtained from all participants.

### Animals

Animal experiments were approved by the IACUC of Shanxi Provincial People's Hospital. Female C57BL/6 mice (6–8 weeks old) were obtained from Sibeifu Biotechnology and housed under standard conditions (12‑h light/dark cycle, 22 ± 2 °C, 55 ± 5% humidity) with free access to food and water. KDM3A⁻/⁻ mice on a C57BL/6 J background were generated by GemPharmatech using CRISPR/Cas9 targeting exon 10 of the Kdm3a‑202 transcript (ENSMUST00000167220.3), which contains a 512 bp coding sequence. sgRNA and Cas9 were microinjected into fertilized eggs, and positive F0 mice were bred to obtain F1 offspring. Genotyping was performed by PCR from tail DNA (Fig. S4).

### Cell culture

Human granulosa cells were isolated from POI patients and healthy controls and cultured in DMEM/F12 supplemented with 10% FBS [6, 59]. The KGN cell line (Procell, CL-0603) was used for overexpression experiments. Mouse granulosa cells were obtained from 8‑week‑old C57BL/6 ovaries, digested with 0.1% hyaluronidase for 5 min at 37 °C, filtered, and cultured in DMEM/F12 with 10% FBS and 1% penicillin‑streptomycin. Adherent cells were used after 24 h [57, 60].

### POI mouse model and AAV treatment

To induce POI, mice received intraperitoneal cisplatin (5 mg/kg) on days 1 and 2; controls received vehicle (DMSO in saline). Model establishment was confirmed by estrous cycle assessment and ovarian histology two weeks later. Seven days after cisplatin, mice received local ovarian injection (10 µL) of AAV2/9 vectors (AAV‑NC, AAV‑KDM3A, or AAV‑ABCA7; 1 × 10^12^ VG/mL) expressing full‑length mouse Kdm3a or Abca7 under the CMV promoter (HanBio Therapeutics). Body weight and health were monitored daily. Ovaries were collected at endpoint for analysis.

### Fertility assessment

Female mice (*n* = 6/group) were mated with fertile males (2:1 ratio) for 3 months starting two weeks after AAV injection. Litter number, pups per litter, and productive female rate were recorded. Average litter size was calculated from three consecutive litters per female.

### Histology and immunohistochemistry

Ovaries were fixed in 4% paraformaldehyde, paraffin‑embedded, and sectioned at 4 μm for H&E staining (Solarbio). Follicle counts and morphology were assessed blindly [1]. For immunohistochemistry, 5‑μm sections were treated with 3% H₂O₂, subjected to antigen retrieval in citrate buffer (pH 6.0), permeabilized with 1% Triton X‑100, and blocked with 5% BSA. Sections were incubated with primary antibodies against FSHR (1:200), CYP19A1 (1:200), or cleaved caspase‑3 (1:200), followed by HRP‑conjugated secondary antibodies and DAB visualization.

### Immunofluorescence staining

Frozen sections or cells were incubated overnight at 4 °C with primary antibodies: anti‑KDM3A (1:100, Proteintech), anti‑AMH (1:50, Santa Cruz), anti‑GDF9 (1:200), anti‑BMP15 (1:200), anti‑DDX4 (1:200), anti‑ABCA7 (1:200), or anti‑Ki67 (1:100). After washing, samples were incubated with Alexa Fluor 488‑ or 594‑conjugated secondary antibodies and counterstained with DAPI. Fluorescence was visualised under a Leica microscope and quantified by ImageJ.

### ChIP‑seq and ChIP‑qPCR

ChIP was performed using a Gene Create Kit with antibodies against KDM3A and H3K9me2 (rabbit IgG as control). Cells (1 × 10⁷) were crosslinked, lysed, and sonicated to 200–1000 bp fragments, followed by immunoprecipitation. DNA was purified and subjected to paired‑end sequencing on an Illumina NovaSeq 6000. Sequencing data were processed with Trimmomatic, Bowtie2, Picard, DeepTools, and IGV. Peaks were called with MACS3, annotated with Homer/ChIPseeker, and analysed for motif enrichment. GO and KEGG analyses were performed using KOBAS (adjusted *p* < 0.05). ChIP‑qPCR primers for the ABCA7 promoter were: forward 5′‑CCTCAACCAACCAAGCAGT‑3′; reverse 5′‑GCAGAGACCAGGGACAGC‑3′.

### Western blotting

Total protein was extracted in RIPA buffer, quantified by BCA assay, separated by SDS‑PAGE, and transferred to PVDF membranes. Membranes were blocked with 5%–7% non‑fat milk and probed overnight with primary antibodies against KDM3A, AMH, PTEN, Bcl‑2, Bax, PGC‑1α, Mfn1, Mfn2, Drp1, OPA1, and GAPDH (all from Proteintech or CST). After TBST washing, membranes were incubated with HRP‑conjugated secondary antibodies, and signals were visualised by ECL and quantified with ImageJ.

### Quantitative real-time PCR

Total RNA was extracted with TRIzol, and 1 μg was reverse‑transcribed using a Vazyme kit. qPCR was performed on a CFX Connect™ system with GAPDH as internal control. Cycling conditions: 95 °C for 10 min, followed by 40 cycles of 95 °C for 10 s, 58 °C for 30 s, and 72 °C for 30 s, with melt curve analysis. Gene expression was calculated by the 2⁻ΔΔCt method. Primer sequences are provided in Supplementary Table S1.

### Mitochondrial function assays

OCR was measured using an XFp Extracellular Flux Analyzer with injections of oligomycin, FCCP, and rotenone/antimycin A; results were normalised to cell number. ATP levels were determined by an ATP assay kit (Beyotime). mtDNA copy number was quantified by qPCR as the ratio of cytochrome B (mtDNA) to β‑globin (nuclear DNA). ROS levels were assessed by DCFH‑DA staining, and mitochondrial membrane potential (ΔΨm) by JC‑1 staining with flow cytometry. MDA, SOD, and GSH were measured using commercial kits (Jiancheng).

### Apoptosis and cell viability assays

Apoptosis was detected by flow cytometry using the Annexin V‑FITC/PI kit (BD Biosciences) or by TUNEL assay (Promega) on paraffin sections. Cell viability was assessed by CCK‑8 assay (Meilunbio): cells were seeded in 96‑well plates, treated, incubated with CCK‑8 for 1 h, and absorbance measured at 450 nm.

### Transmission electron microscopy

Granulosa cells were fixed in 1% osmium tetroxide, dehydrated in graded ethanol and acetone, embedded in Epon‑812, sectioned, stained with uranyl acetate and lead citrate, and examined under a Hitachi HT7800 TEM.

### GEO data analysis and statistics

GSEA was performed on GSE84078 data using clusterProfiler and org.Mm.eg.db. Spearman correlation analysis (psych package) assessed associations between ABCA7 and mitochondrial genes. Statistical analyses were performed with GraphPad Prism 9.0. For small samples (n ≤ 6), non‑parametric tests (Mann–Whitney U for two groups; Kruskal–Wallis with Dunn's post‑hoc for multiple groups) were used. For clinical data, normality was assessed by Shapiro–Wilk; parametric tests (t‑test or ANOVA with Tukey's post‑hoc) were applied when assumptions were met, otherwise non‑parametric tests. Pearson correlation was used for KDM3A vs. ovarian reserve markers. *P* < 0.05 was considered significant. All experiments were repeated at least three times.

### Statistical analysis

Statistical analyses were performed using GraphPad Prism 9.0 (GraphPad Software, USA). Data are presented as mean as mean ted as mean as meaolving small sample sizes (n ≤ 6 per group, e.g., animal experiments), non-parametric tests were applied: the Mann–Whitney U test for two-group comparisons and the Kruskal–Wallis test followed by Dunn’s post-hoc test for multiple-group comparisons. For clinical data with larger sample sizes, normality was assessed using the Shapiro–Wilk test; parametric tests (unpaired t-test or one-way ANOVA with Tukey’s post-hoc test) were used when data met the assumptions of normal distribution and homogeneity of variance; otherwise, the corresponding non-parametric tests were used. Spearman correlation analysis was used to evaluate associations between ABCA7 and other genes, with correlation coefficients (cor) and *p*-values reported. Genes with |cor|> 0.3 and *p* < 0.05 were selected for further analysis. Pearson correlation and linear regression analyses were performed to assess the relationship between KDM3A expression and clinical ovarian reserve markers. A *P* value < 0.05 was considered statistically significant. Each experiment was repeated at least three times.

## Supplementary Information

Supplementary Material 1.

## Data Availability

All the raw datasets generated during the current study are available from the corresponding author on reasonable request.

## Abbreviations

ABCA7: ATP-binding cassette sub-family A member 7
AMH: Anti-Müllerian hormone
ChIP-seq: Chromatin immunoprecipitation sequencing
DDP: Cisplatin (used as abbreviation in figures/text)
Drp1: Dynamin-related protein 1
FSHR: Follicle-stimulating hormone receptor
GDF9: Growth differentiation factor 9
KDM3A: Lysine-specific demethylase 3A (also known as JMJD1A)
KGN: Human granulosa-like tumour cell line
Mfn1: Mitofusin 1
OPA1: Optic atrophy 1
PGC-1α: Peroxisome proliferator-activated receptor gamma coactivator 1-alpha
POI: Premature ovarian insufficiency
SASP: Senescence-associated secretory phenotype
