# Supplementary material for: Lysine-specific demethylase 3A (KDM3A) protects against ovarian dysfunction in premature ovarian insufficiency by transcriptional activation of ATP-binding cassette sub-family A member 7 (ABCA7)-mediated mitochondrial homeostasis
Source: Mol Biomed. 2026 Sep 8;7:165. doi: 10.1186/s43556-026-00567-5 (PMC13550290; doi:10.1186/s43556-026-00567-5)
Supplement: Supplementary file 1 [file 43556_2026_567_MOESM1_ESM.docx]

**Lysine-specific demethylase 3A (KDM3A) protects against ovarian dysfunction in premature ovarian insufficiency by transcriptional activation of ATP-binding cassette sub-family A member 7 (ABCA7)-mediated mitochondrial homeostasis**

Xiangrong Cui^1,2†^, Chongyang Han^5^^†^, Puhua Zhang^2^, Ruixiang Zhu^3^, Li Peng^4^, Jiali Luo^3^, Chenyu Jia^2^, Ruotong Ju^2^, Xinyu Zhu^3^, Shu Wang^3^, Huihui Li^2^, Xiaochun Liu^1*^, Xuan Jing^3^^*^

1 Third Hospital of Shanxi Medical University, Shanxi Bethune Hospital, Shanxi Academy of Medical Sciences, Tongji Shanxi Hospital, Taiyuan, 030032, China

2 Department of Reproductive Medicine Center, Children's Hospital of Shanxi, The Affiliated Children's Hospital of Shanxi Medical University, Shanxi Maternal and Child Health Hospital, Taiyuan, 030001, China.

3 Department of Clinical Laboratory of Shanxi Provincial People's Hospital, Shanxi Medical University, Taiyuan, 030001, China.

4 Shanxi University of Traditional Chinese Medicine; Taiyuan, Shanxi, 030001, China.

5 Department of Nephrology of Shanxi Provincial People's Hospital, Shanxi Medical University, Taiyuan, 030001, China.

^†^Xiangrong Cui, and Chongyang Han contributed equally to this work.

*Corresponding author: Xuan Jing ([jx05070103@163.com](mailto:jx05070103@163.com)); Xiaochun Liu ([liuxiaochun@sxbqeh.com.cn](mailto:liuxiaochun@sxbqeh.com.cn))

**Supplementary Figure S1**

**Supplementary Figure S1 | Baseline ovarian phenotype of KDM3A⁻/⁻ mice under untreated conditions.**

Comparison of ovarian parameters between untreated KDM3A⁺/⁺ (wild‑type) and KDM3A⁻/⁻ (knockout) mice. a, Ovarian index (ovary weight/body weight, mg/g). b, Ovarian volume (mm³). c, Serum AMH levels. d, Serum FSH levels. e, Serum LH levels. f, Serum E₂ levels. g–j, Follicle counts: primordial (g), primary (h), secondary (i), and antral (j) follicles. No significant differences were observed between KDM3A⁺/⁺ and KDM3A⁻/⁻ mice for any of the parameters measured (ns, not significant). Data are presented as mean ± SEM (n = 6 mice per group). Statistical comparisons were performed using the Mann–Whitney U test.

**Supplementary Figure S2**

**Supplementary Figure S2 | KDM3A overexpression in KGN cells and dose‑dependent effects of cisplatin on cell viability.**

a, Representative western blot confirming KDM3A overexpression in KGN cells transfected with Ad‑KDM3A compared with Ad‑NC (negative control) and untreated control. GAPDH served as the loading control. b, Quantification of KDM3A protein levels normalized to GAPDH (n = 3 independent experiments). c, Dose‑dependent effects of cisplatin (DDP) on KGN cell viability after 24 h of treatment. Cells were treated with increasing concentrations of DDP (0–10 μg/mL). d, Dose‑dependent effects of cisplatin (DDP) on KGN cell viability after 48 h of treatment (0–10 μg/mL). Cell viability was assessed by CCK‑8 assay and expressed as percentage of untreated control. Data are presented as mean ± SEM (n = 3 independent experiments). *P < 0.05, **P < 0.01, ***P < 0.001 vs. untreated control. Statistical comparisons were performed using one‑way ANOVA with Tukey's post‑hoc test.

**Supplementary Figure S3**

**Supplementary Figure S3 | Validation of ABCA7 overexpression in ovarian tissues.**

a, ABCA7 mRNA levels in ovarian tissues from Control, POI, and POI+AAV-ABCA7 groups measured by qRT-PCR (n = 6 mice per group). b, Representative western blot of ABCA7 protein expression in ovarian tissues from the indicated groups. GAPDH served as the loading control. c, Quantification of ABCA7 protein levels normalized to GAPDH (n = 6 mice per group). AAV-ABCA7 treatment significantly increased both ABCA7 mRNA and protein levels in the ovaries of POI mice compared with the POI group. Data are presented as mean ± SEM. ***P < 0.001; ns, not significant. Statistical comparisons among multiple groups were performed using the Kruskal–Wallis test followed by Dunn's post-hoc test.

**Supplementary Figure S4**

**Supplementary Figure S1 | Genotyping strategy for KDM3A knockout mice.**

Schematic diagram showing the genotyping strategy for KDM3A⁻/⁻ mice. The targeting strategy for exon 10 of the Kdm3a gene (transcript ENSMUST00000167220.3) is illustrated. The positions of the forward (F1, F2) and reverse (R1, R2) genotyping primers are indicated by arrows. The wild-type and knockout (KO) alleles are distinguished by PCR amplification, with the KO fragment generated from the targeted allele. Primer sequences are as follows: Forward1: 5′-TGGCTAGGGCAGAGAACTCTAGTATG-3′; Reverse1: 5′-CAAAGGTTACCTTCCTGTA AGAGC-3′; Forward2: 5′-TCTGAGTGAGCCCAAGGTAGC-3′; Reverse2: 5′-TAAGTCAGAG TATGGAGTCCCGTGC-3′. The expected PCR products for the wild-type and mutant alleles are indicated.

**Supplementary Table S1** Sequences of primers used for fluorescence quantitative PCR in this study. Annealing temperature for all primers was 58°C.

| Gene | Primer sequence (forward) | Primer sequence (reverse) |
| --- | --- | --- |
| Human-KDM3A | GTGCTCACGCTCGGAGAAA | GTGGGAAACAGCTCGAATGGT |
| Human-GAPDH | AATGGATTTGGACGCATTGGT | TTTGCACTGGTACGTGTTGAT |
| Mus-KDM3A | GTGGGGAAGCGATTCCTCAG | GTCTTTCTTAGTAACGTCGGTGT |
| GDF9 | GGCACAAAGGTTCAGGGGG | CACCCGGTCCAGGTTAAACA |
| AMH | CCACACCTCTCTCCACTGGTA | GGCACAAAGGTTCAGGGGG |
| BMP15 | TGGGGAGTGGTGCTTTTTATG | GGGCAATGTAGGGTCGTCAG |
| DDX4 | GAGAACACATCTACAACTGGTGG | CCTCGCTTGGAAAACCCTCT |
| PTEN | TGGATTCGACTTAGACTTGACCT | GCGGTGTCATAATGTCTCTCAG |
| FSHR | TGCTCTAACAGGGTCTTCCTC | TCTCAGTTCAATGGCGTTCCG |
| CYP19A1 | AACCCCATGCAGTATAATGTCAC | AGGACCTGGTATTGAAGACGAG |
| IL-18 | GTCACAGCCAGTCCTCTTACTTCAC | CCCTTGTCGAGAATGGGCAG |
| IL-1β | GACCAGAATGTGCCACGGTT | CTCGCCTGCTCATCAACAAG |
| IL-6 | GTTCTCTGGGAAATCGTGGA | GGAAATTGGGGTAGGAAGGA |
| TNF-α | TATGGCCCAGACCCTCACA | GGAGTAGACAAGGTACAACCCATC |
| CCL-2 | TTCTTCGATTTGGGTCTCCTTG | TTCTTCGATTTGGGTCTCCTTG |
| ICAM1 | GTGATGCTCAGGTATCCATCCA | CACAGTTCTCAAAGCACAGCG |
| MMP3 | GGCCTGGAACAGTCTTGGC | TGTCCATCGTTCATCATCGTCA |
| Mus-GAPDH | AGGTCGGTGTGAACGGATTTG | TGTAGACCATGTAGTTGAGGTCA |
